# Automated Global Positioning Layout *(GPL)* for accuracy assessment in CAD-CAM mandibular reconstruction – method introduction

**DOI:** 10.1038/s41598-026-44238-5

**Published:** 2026-05-15

**Authors:** Elisa Vargiu, Giorgia Menapace, Giordana Bettini, Laura Tognin, Francesca Uccheddu, Giorgia Saia, Giorgio Bedogni, Roberto Meneghello, Alberto Bedogni

**Affiliations:** 1 Department of Management and Engineering, University of Padua, Padova, Italy; 2 Unit of Maxillo-Facial Surgery, Head and Neck Department, University Hospital of Parma, Parma, Italy; 3 Maxillofacial Surgery Unit, ‘‘S. Anna’’ Hospital, Como, Italy; 4 Department of Industrial Engineering, University of Padua, Padova, Italy; 5 Department of Neuroscience, Unit of Maxillofacial Surgery, University of Padua, Padova, Italy; 6 Department of Medical and Surgical Sciences, Alma Mater Studiorum- University of Bologna, Bologna, Italy; 7 Department of Primary Health Care, Internal Medicine Unit addressed to Frailty and Aging, “S. Maria delle Croci” Hospital, AUSL Romagna, Ravenna, Italy; 8 Regional Center for the Prevention, Diagnosis, and Treatment of Medication and Radiation-Related Bone Diseases of the Head and Neck, Hospital Trust of Padova, Padova, Italy

**Keywords:** Mandibular reconstruction, Patient-specific implant, Computer-assisted surgery, Computer-aided design, Computer-aided manufacturing, Assessment of accuracy, Automatization, Computational biology and bioinformatics, Engineering, Health care, Medical research

## Abstract

Assessing accuracy in CAD-CAM mandibular reconstruction poses significant challenges but is essential for ensuring reliable outcomes. Existing methods are often operator-dependent, lacking repeatability and reproducibility. This study introduces the Global Positioning Layout (GPL) method, an accuracy assessment technique integrated into the reconstruction protocol based on CAD-CAM and additive printing technology. We describe its methodology and present its implementation through an automated workflow. Key principles of accuracy assessment were identified and structured as Requirements, Data input, Data reference system, and Data output. The necessary 3D virtual models were defined: planned mandible, reference mandible, patient-specific implant (PSI), postoperative mandible, and postoperative PSI. A unique coordinate system (GPL-RS) was built on the reference mandible. Three Roto-Translational Matrices (RTMs) were applied to measure movements and deviations between the designed and postoperative models to assess reconstruction accuracy. Five clinical cases with different operational diseases and defects were analysed, comparing spatial deviations between manual and automated methods. The GPL method represents a promising advancement in assessing the accuracy of CAD-CAM reconstructions, providing valuable insights that can improve surgical outcomes.

## Introduction

Computer-aided design and manufacturing (CAD-CAM) is an emerging technology in head and neck reconstructive surgery, providing patient-specific devices to restore facial symmetry and volumes^1^. The introduction of additive manufacturing and 3D printing has revolutionized the planning of resections and reconstructions in complex cases^2,3^. Virtual surgical planning (VSP) offers a 3D visualization of patient anatomy for personalized surgical planning^4^. Computer-assisted surgery (CAS) enhances precision and reduces the surgeon’s learning curve^5^. CAD-CAM technology also provides objective data to ensure consistency. However, VSP may increase patient expectations before surgery^6^. Despite these advancements, evaluating the accuracy of CAD-CAM mandibular reconstruction is challenging, with no standardized protocols for VSP, virtual design, or additive printing in customized reconstructive surgery^7^. To optimize outcomes, CAS performance should be quantitatively assessed^8,9^. To date, several methods have been developed to assess CAD-CAM mandibular reconstruction accuracy, including comparisons of 2D CT images and 3D CT scans^10^. Some methods rely on linear and angular measures based on the distance between anatomical landmarks. Such landmarks are drawn as single points on two-dimensional CT images or 3D virtual model surfaces^11–17^. These methods, however, are operator-dependent and may lack repeatability. A guideline has been recently proposed to standardize evaluation methods, including strategies for imaging, defect classification, data comparison, and volume assessment of 3D models^18^.

To address this challenge, this study aims to establish metrological principles for computer-aided accuracy assessment in CAD-CAM mandibular reconstruction and to introduce the GPL method, presenting its automation.

## Methods

### Study setting

The GPL method was developed at the Departments of Neuroscience-DNS and of Management and Engineering of the University of Padova (Italy). This study was conducted in full accordance with the principles outlined in the Declaration of Helsinki.

### Basic principles of accuracy assessment

The basic principles of accuracy assessment are listed as follows:

A. Requirements:
  - Functionality: computing of spatial relationships between the planned mandibular reconstruction and the postoperative result.
  - Independency: the measurement framework must be independent of the specific software platform used, and the results must be independent of the operator (Operator uncertainty principle), therefore minimizing human error and measurement variability.
  - Compatibility: input and output data must be given in a format suitable for any CAD-based system.
  - Generality: the methodology must be applicable to different types of mandibular defects and reconstruction procedures, enabling broad clinical applicability.
  - Rigid workpiece: any patient-specific device is a rigid part of infinite stiffness or whose distortion does not exceed specified tolerances by applying pressure or forces during and after standard surgery. It provides a stable reference for accuracy assessment.
B. Data input:
  - any kind of mandibular bone defect.
  - any kind of VSP of mandible reconstruction.
  - any kind of CAD patient-specific device.
C. Data reference system:
  - a defined and unique, intrinsic, 3D coordinate system (X-Y-Z), called “reference system”, is used to describe the spatial position and orientation of any model.
D. Data output:
  - a three-dimensional assessment of spatial errors, according to the reference system.
  - errors concern the position and orientation of the patient-specific device.

### Definition of operational models for GPL applications

The Digital Imaging and Communications in Medicine (DICOM) data obtained from the preoperative CT scans are imported into a given virtual planning software and converted into surface models in a Standard Tessellation Language (STL) file format.

The virtual 3D model (i.e. CAD model) of the facial skeleton is segmented to obtain the “*native mandible*”, which consists of the entire mandible, including both the diseased and healthy parts. The native mandible is used to extract the “*planned mandible”*, which consists of the healthy portion of the native mandible devoid of the diseased bone.

For bone defects limited to half of the mandible, the native mandible is mirrored and fitted to obtain the *“reference mandible”*. In case of gross deformation of the mandible exceeding the midline, the reference mandible is obtained through superimposition, scaling, and fitting of healthy 3D models of the mandible taken from a virtual image library of lower jaws.

Then, the reference mandible is used to design the patient-specific implant (PSI). The final virtual 3D model of the device is called “*designed PSI”*. The combination of the planned mandible and the designed PSI is called the “*designed model”.*

**Fig. 1:**
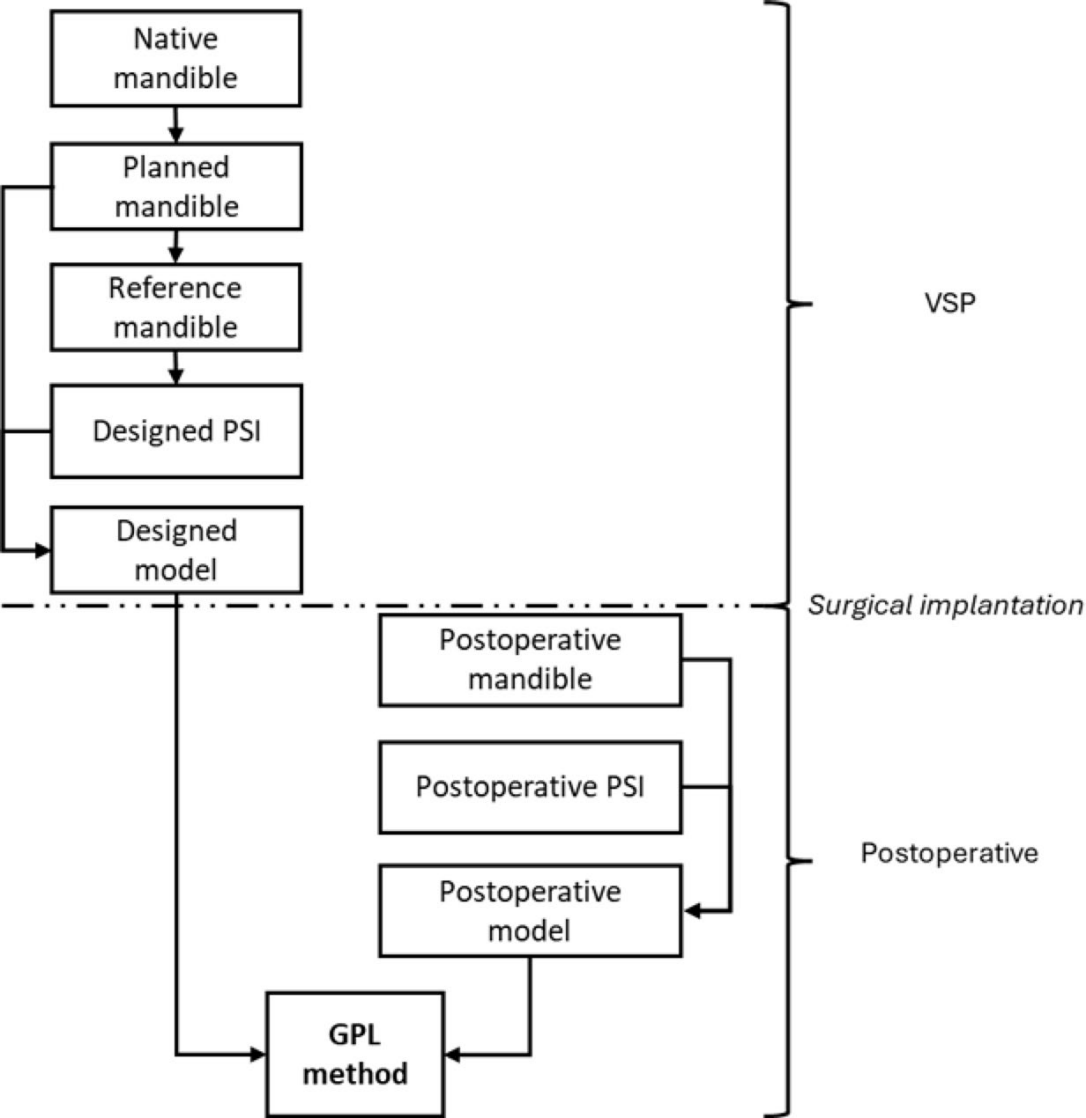
Sequence of models obtained from the Virtual Surgical Planning and the Postoperative follow-up.

The virtual 3D model of the postoperative result is obtained from the postoperative CT scans using the same approach and is called the “*postoperative model”*. This latter consists of the combination of two features: *the “postoperative mandible”*, which is the remaining portion after surgical resection of the diseased bone volume, and *the “postoperative PSI”*, which is the patient-specific implant following surgical implantation. The sequence of the operational models is depicted in Fig. 1:

In conclusion, the following virtual 3D models are required to apply the GPL method: planned mandible, reference mandible, designed PSI, postoperative mandible, and postoperative PSI.

### GPL data coordinate reference system

In the GPL method, the virtual 3D models of the VSP are positioned and aligned in a unique coordinate (X-Y-Z) reference system (GPL-RS). GPL-RS is based on the reference mandible through an automated process of identification of specific geometric features (see § Step 2: Reference system (GPL-RS) definition).

The virtual 3D postoperative model is positioned and oriented in a coordinate reference system that originates from CT data acquisition. Therefore, alignment of the virtual 3D postoperative model to GPL-RS is essential to perform the analysis and comparison according to the GPL methodology.

### GPL workflow

**Fig. 2:**
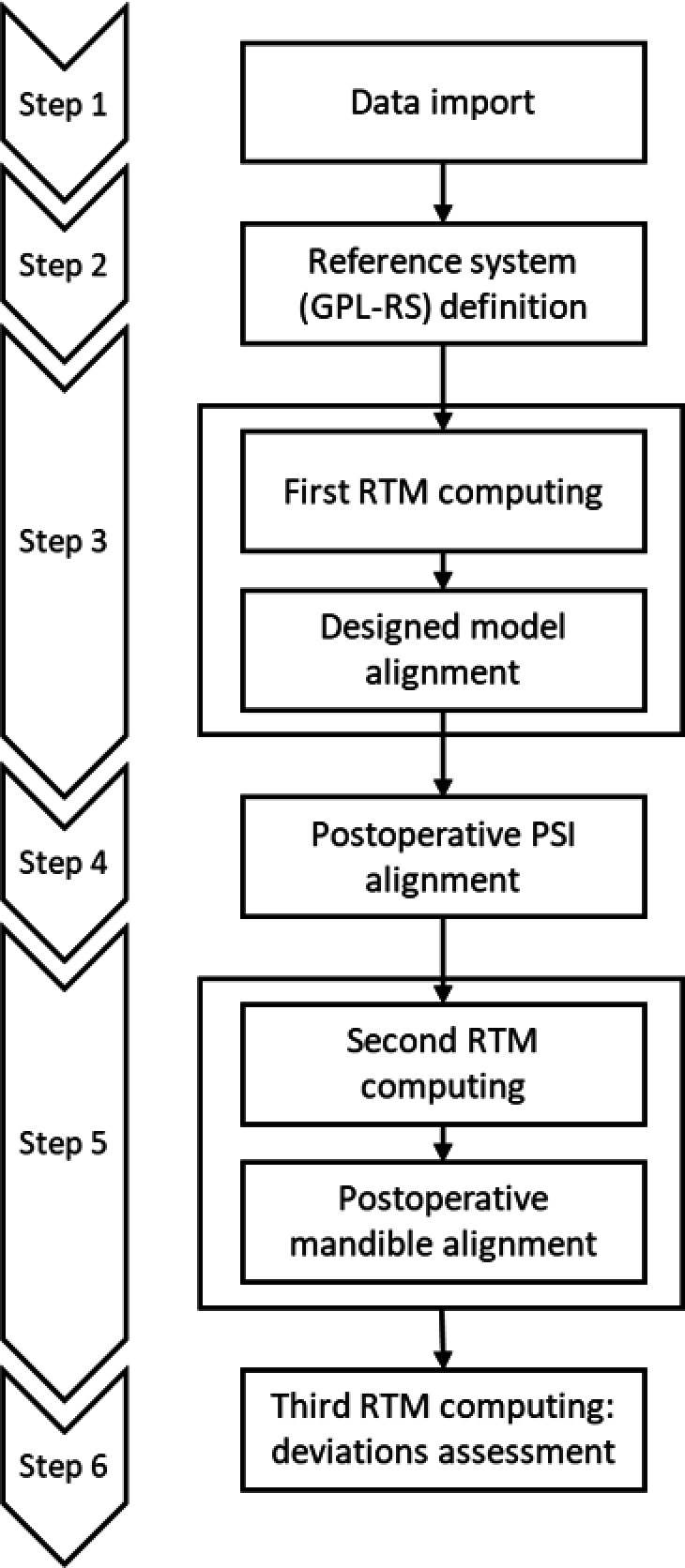
Global Positioning Layout workflow.

The GPL workflow is depicted in Fig. 2:

In the following, a brief description of the main steps is presented:

Step 1: Data import.

**Fig. 3:**
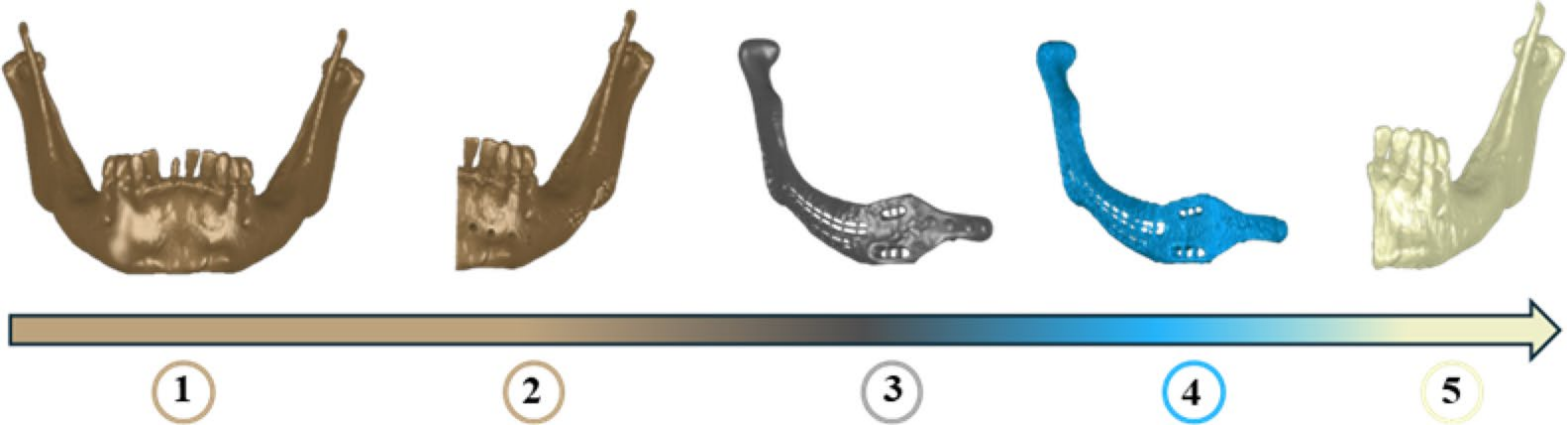
STL models imported into the software: (1) planned mandible (2) reference mandible (3) design prosthesis (4) postoperative prosthesis (5) postoperative mandible.

Five 3D virtual models are imported into the application software: (a) planned mandible, (b) reference mandible, (c) designed PSI, (d) postoperative PSI, and (e) postoperative mandible (Fig. 3).

Step 2: Reference system (GPL-RS) definition.

The GPL-RS is constructed on the reference mandible.

**Fig. 4:**
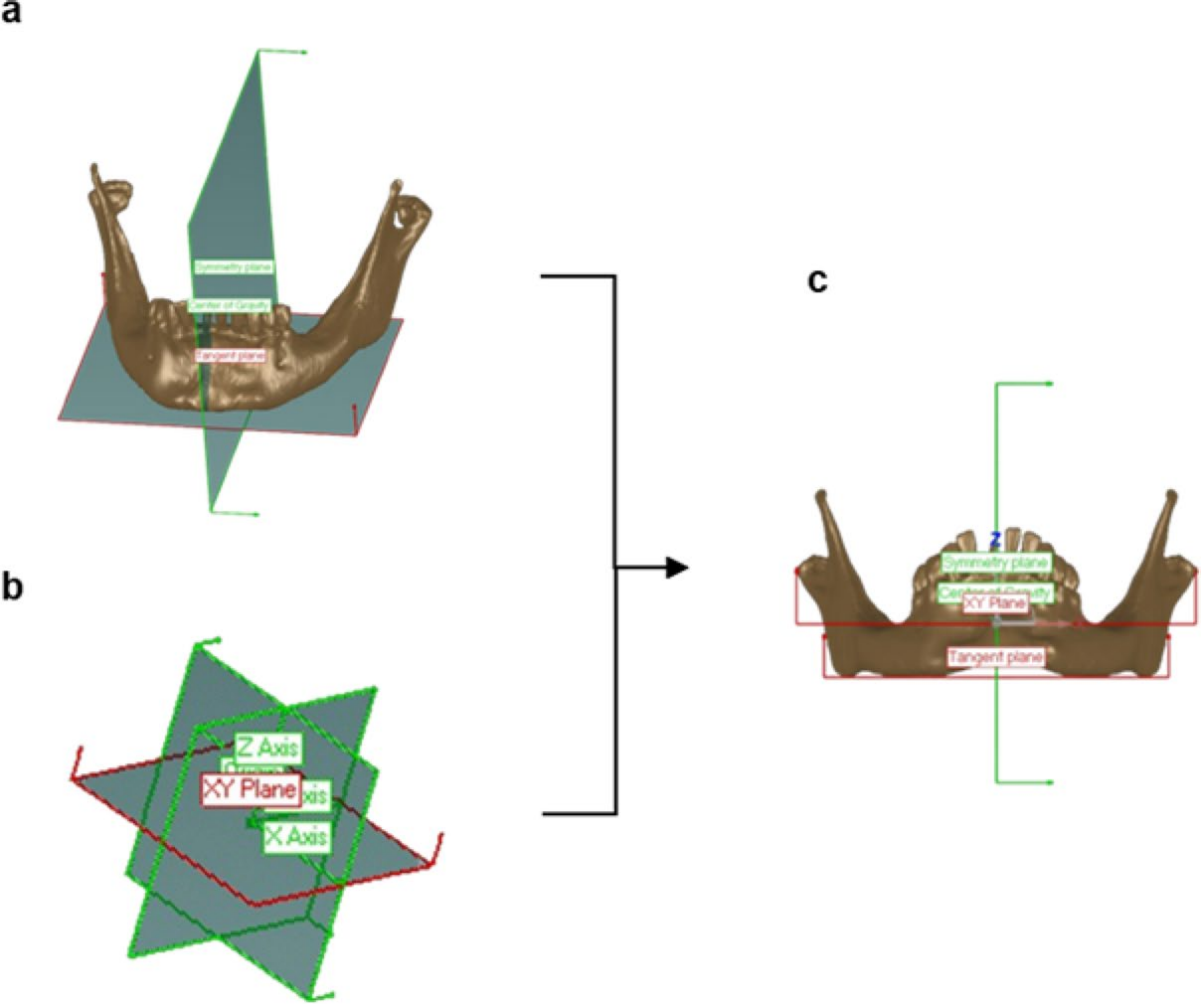
Reference system (GPL-RS) definition and first RTM computing. (**a**) Intra-mandibular geometric features. (**b**) Common coordinate reference system. 4c: Reference mandible alignment.

In brief, 3 intra-mandibular geometric features are computed: the *centre of gravity (i.e. barycentric point)*, a *symmetry plane*, and a *plane tangent to the inferior edge of the mandible* (Fig. 4.A).

The application software computes the centre of gravity of the object.

The tangent plane to the inferior border of the mandible is then defined so as to coincide with one triangular facet of the object’s convex hull, thereby minimizing its distance from the centre of gravity.

The symmetry plane of the reference mandible - intentionally constrained to pass through the centre of gravity - is subsequently constructed orthogonally to this tangent plane by using the vector product between its normal vector and the direction vector of the tangent plane.

To define the GPL-RS, the intra-mandibular geometric features are then associated with the common coordinate reference system (Fig. 4.B) following the ordered sequence:

1. centre of gravity → OO origin of axes.
2. symmetry plane → YZ plane.
3. tangent plane to inferior mandibular edge → XY plane.

This sequence aligns (i.e. translates and rotates) the *reference mandible* onto the GPL-RS coordinate system (Fig. 4.C).

Step 3: First roto-translational matrix (RTM) computing and designed model alignment.

The quantitative estimation of the above-mentioned movements of the reference mandible is described by 3 rotational and 3 translational components according to the X, Y, and Z axes of the GPL-RS, which define the 1st RTM: positive rotation angles cause a counterclockwise rotation around the axes while positive translations cause a movement along the axes.

Below is provided the general form of the roto-translational matrix:

1 $$RTM=\left[\begin{array}{cccc}{r}_{11}&{r}_{12}&{r}_{13}&{t}_{x}\\{r}_{21}&{r}_{22}&{r}_{23}&{t}_{y}\\{r}_{31}&{r}_{32}&{r}_{33}&{t}_{z}\\0&0&0&1\end{array}\right]$$

It’s a 4 × 4 matrix where the upper-left 3 × 3 sub-matrix represents the rotation matrix, and the last column (3 × 1) of the matrix represents the translational vector.

**Fig. 5:**
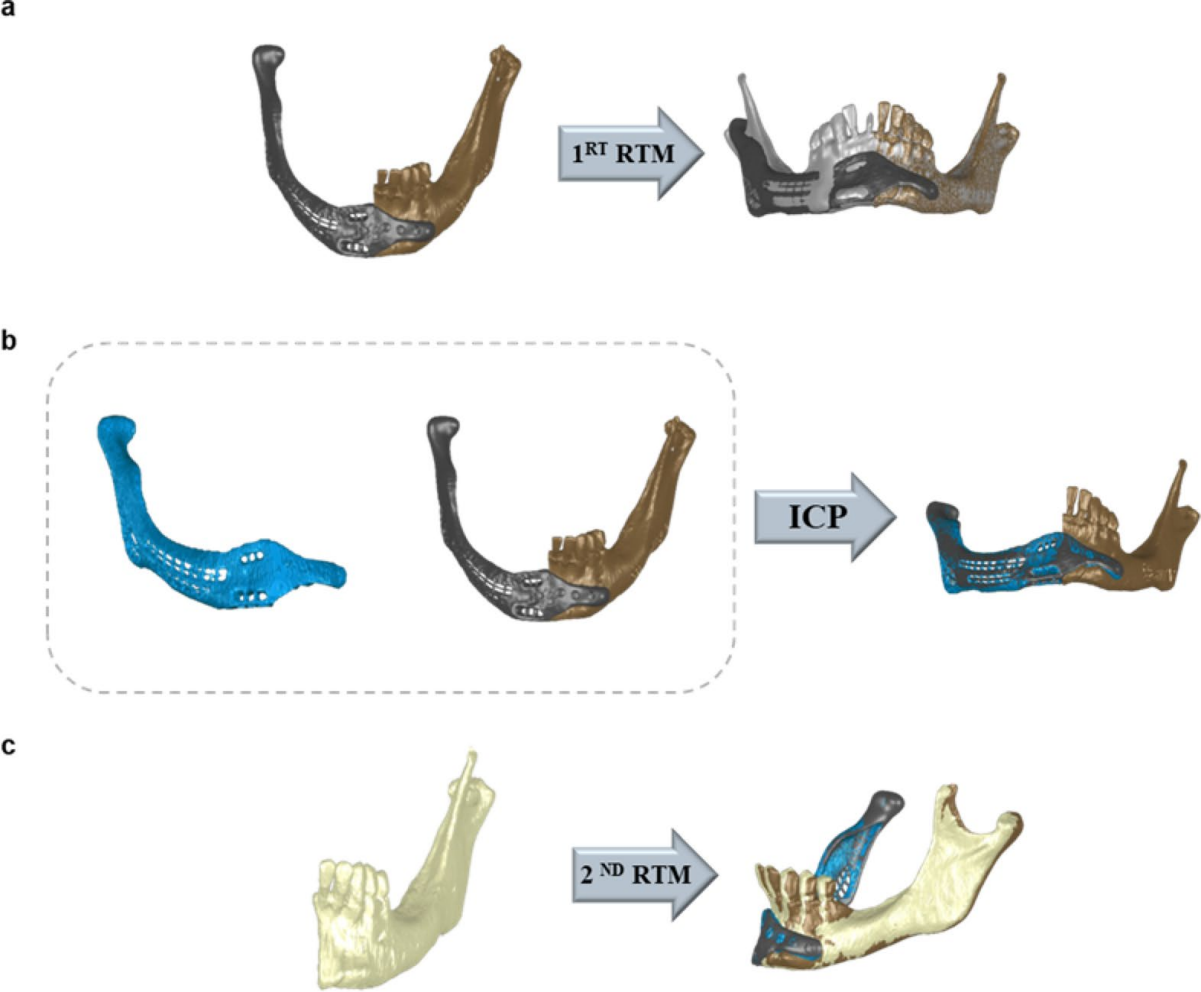
Alignment steps. (**a**) Application of the first roto-translational matrix for the alignment of the designed model (planned mandible and designed prosthesis) on the GPL-RS, (**b**) Alignment of the postoperative prosthesis with the designed prosthesis, (**c**) Application of the second roto-translational matrix to the postoperative mandible. The displacement between the two mandibular stumps quantifies the deviation between the preoperative and the postoperative results.

The 1st RTM is then applied to align *the designed model* (*planned mandible* + *designed PSI)* to the GPL-*RS (*Fig. 5.*a)*.

Step 4: Postoperative PSI alignment.

The *postoperative PSI* is then aligned to the *designed PSI* through two consecutive steps using ICP algorithms (Fig. 5.b). The initial step involves the superimposition of the prosthetic model onto the design model. This is achieved using a best-fit method that minimizes the distance between the two models by automatically selecting corresponding points. In the second step, a more detailed ICP-based alignment is employed to improve the preliminary superimposition.

Step 5: Second roto-translational matrix (RTM) computing and postoperative mandible alignment.

The quantitative estimation of the *postoperative PSI* movements is the 2nd RTM. The 2nd RTM is then applied to align the *postoperative mandible* to the *GPL*-*RS (*Fig. 5.*c)*.

Step 6: Third roto-translational matrix (RTM) computing: measure of deviations.

For the assessment of the accuracy in CAD-CAM mandibular reconstruction, the computation of the deviation between the postoperative model and the designed model requires the superimposition of the postoperative mandible onto the designed mandible.

Similarly to the procedure conducted for prosthetic models, it is necessary to undergo two alignment phases.

The quantitative estimation of the *postoperative mandible* movements is the 3rd RTM.

The 3rd RTM represents the deviations (rotational and translational errors) and measures the accuracy of the reconstruction. The general form of the roto-translational matrix is given by Eq. 1.

The rotational movements along the X, Y, and Z axes performed by the mandible in the postoperative follow-up can be computed employing Euler’s formulas:

2 $${\theta}_{x}=\mathrm{a}\mathrm{tan}2(-{r}_{23},{r}_{33})$$

3 $${\theta}_{y}=\mathrm{a}\mathrm{sin}\left({r}_{13}\right)$$

4 $${\theta}_{z}=\mathrm{a}\mathrm{tan}2(-{r}_{12},{r}_{11})$$

The translations, instead, can be directly extracted from the roto-translational matrix.

5 $$T=\left[\begin{array}{c}tx\\ty\\tz\end{array}\right]$$

### Automated GPL method

The methodology has been automated using the Python programming tool integrated within Geomagic Wrap^®^ 2021 (Oqton Inc., South Carolina, US). To implement the necessary functions, the script utilizes both the software’s internal GEO libraries and its API. The workflow is initiated when the operator selects and loads the patient models through a dedicated import prompt. Once the models are loaded, the script proceeds to automatically execute all subsequent phases of the GPL method. As the final step, the three roto-translational matrices (RTMs) generated are saved as .tfm files in the folder initially specified by the operator.

### Application of the automated GPL

Five clinical cases (Table 1) presenting two different mandibular defect types (H and HCL)^19^ were analysed. The cases were selected from a prospective patient cohort treated at the Maxillofacial Surgery Unit of the University Hospital of Padua between 2012 and 2017. Patients were consecutively enrolled if they presented mandibular bone defects involving the condylar unit and underwent reconstruction with a PSI. The availability of a postoperative CT, segmented via Mimics software (Materialise, NV, Leuven, Belgium), scan was also required to ensure homogeneity and a consistent anatomical context for assessing the feasibility of the GPL method.

**Table 1:** Patient population.

ID patients	Sex	Defect	Operational diagnosis	Underlying disease
1	M	H	ORN^1^; Chronic radiodermatitis (IV grade)	Personal history of oropharyngeal cancer (SCC)
2	M	HCL	MRONJ^2^	Metastatic prostate cancer
3	F	HCL	MRONJ^2^	Metastatic breast cancer
4	F	H	MRONJ^2^	Metastatic breast cancer
5	F	HCL	Chronic osteomyelitis	None

^1^Osteoradionecrosis, ^2^Medication-related osteonecrosis of the jaws.

**Fig. 6:**
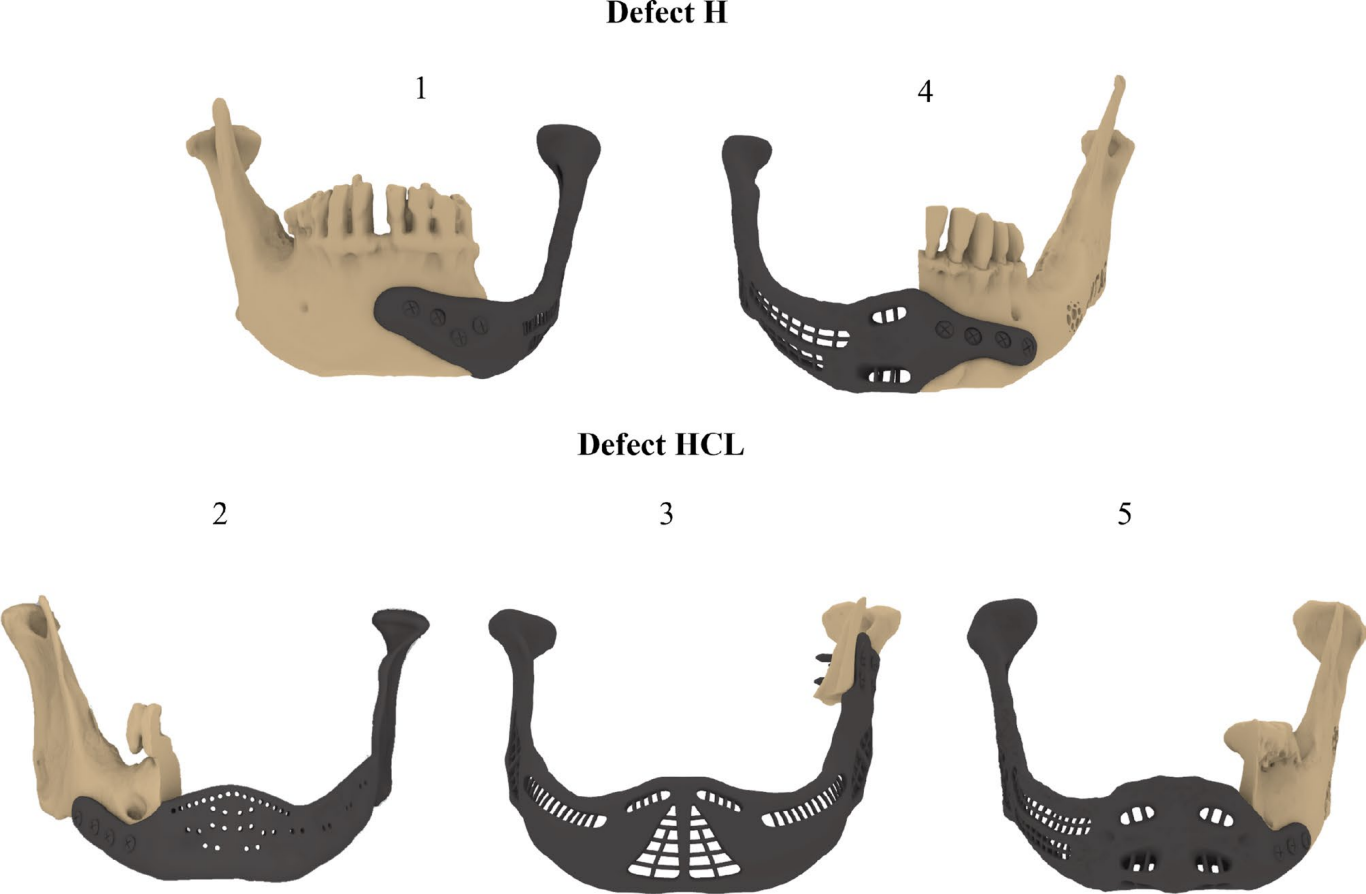
Design overview of the titanium REPLICA prosthesis and application on selected mandibular defects.

All patients underwent mandibular reconstruction using custom-made REPLICA prosthetic devices, whose design and manufacturing process has been detailed in a previous publication from our group^20^. An overview of the titanium REPLICA prosthesis design and its application on selected mandibular defects is presented in Fig. 6.

### Comparative analysis

To assess the performance of the automated methodology, it was compared against the step-by-step manual implementation of the method within the same software. The comparison focused on the third roto-translational matrix (RTM), which quantifies the deviations between the preoperative plan and the postoperative outcome. Both the rotational and translational components of the matrix were examined to identify any significant differences between the two approaches.

## Results

### Application of the automated GPL

The automated methodology was applied to the five patients included in the study. Table 2 presents the results of the third roto-translational matrix.

**Table 2:** Results of the third roto-translational matrix for the automated methodology.

ID patients	Rot-X	Rot-Y	Rot-Z	Trans-X	Trans-Y	Trans-Z
	[Deg]			[mm]		
1	0.972	-1.257	-1.147	0.84	0.158	0.094
2	5.4	-4.573	-2.314	-1.472	-2.197	1.898
3	1.423	-0.165	1.561	1.629	0.323	-1.155
4	-0.126	1.247	1.318	0.287	0.206	0.173
5	2.855	-1.126	1.245	-1.381	-2.456	1.033

For the rotational components, a positive value indicates a counterclockwise rotation relative to the referenced axis, while a negative value indicates a clockwise rotation. For the translational components, a positive value represents a displacement in the same direction as the referenced axis, whereas a negative value represents a displacement in the opposite direction.

### Comparative analysis

**Fig. 7:**
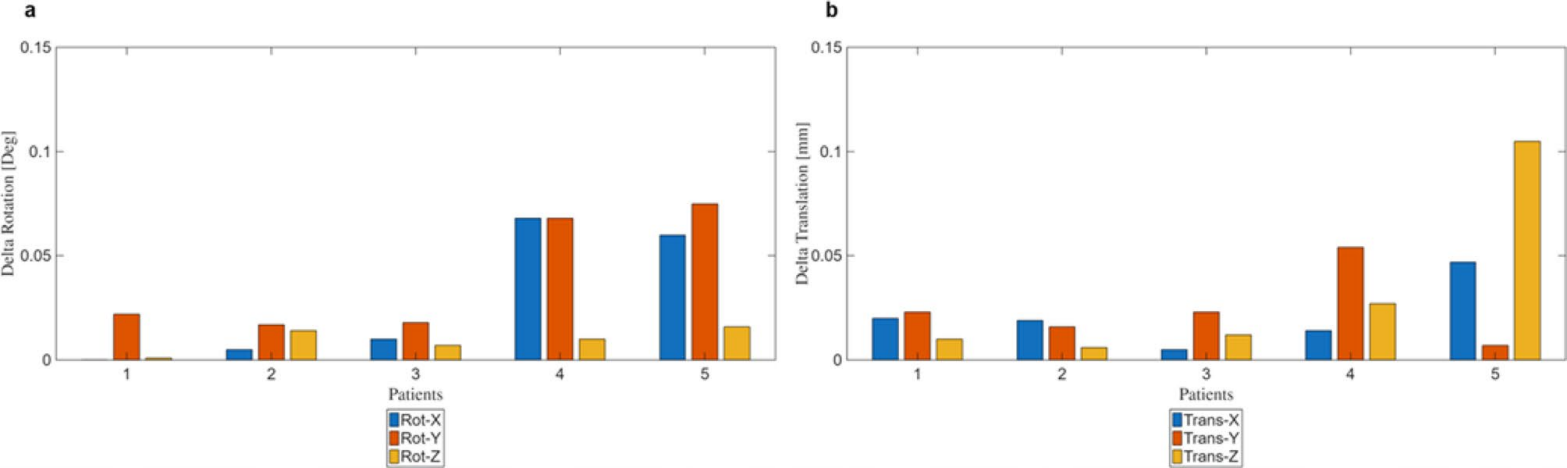
Differences between manual and automated procedures. (**A**) Rotational differences (delta) in X, Y, and Z axes. (**B**) Translational differences (delta) in X, Y, and Z axes.

The results of the comparison between the manual and automated procedures for the third roto-translational matrix are presented in Fig. 7.

Figure 7a displays the rotational differences between the two methods. Across all cases, the results indicate minimal rotational discrepancies, with most errors remaining below 0.02 degrees in the X, Y, and Z directions. This suggests a high degree of accuracy in alignment, particularly for patients 1 to 3, where the differences are negligible.

For Patients 4 and 5, slightly higher rotational discrepancies are observed, particularly in the X and Y components, with maximum errors of 0.068 degrees and 0.075 degrees, respectively. While these values are higher compared to the other cases, they still represent relatively small differences, indicating overall performance of the method.

Figure 7b illustrates the comparison of the three translational components. The most significant errors occur in case 5, particularly in the Z translation component, where the error reaches 0.105 millimetres.

Across all cases, the results indicate that most translational errors remain within an acceptable range, typically below 0.03 millimetres, suggesting a high degree of precision in the alignment of the reconstructed models. The X and Y components generally show minimal deviations, with errors primarily ranging from 0.005 millimetres (in patient 3) to 0.047 millimetres (in patient 5) for the X component, and from 0.007 millimetres (in patient 5) to 0.054 millimetres (in patient 4) for the Y component.

## Discussion

Recently developed computer-assisted reconstructive surgery techniques integrate advanced 3D imaging, computer simulation software, and CAD/CAM technologies^2^. These systems aim to enhance surgical outcomes and ensure reproducibility. The process of computer-assisted surgery (CAS) consists of sequential phases: (1) image data acquisition and elaboration/segmentation, (2) virtual surgical planning (CAD), (3) manufacturing of the final construct (CAM), (4) surgical treatment, and (5) evaluation of the result. Each phase is prone to errors that may impact both the outcome and patients’ quality of life^8^. In line with increasing efforts to standardize and automate key stages of reconstructive surgery^21–24^, this study introduces the Global Positioning Layout (GPL) method, presenting its basic principles and its automated workflow for comparing distortions between the postoperative and planned virtual models.

Unlike using the native mandible as a reference, the GPL relies to the planned model, avoiding alterations due to underlying pathological conditions. This approach aligns with the views of other authors^8,11,25,26^.

A key feature of GPL is its assumption that the titanium device used in the reconstruction maintains its geometry post-implantation. By first superimposing the titanium device, the method provides a consistent basis for accuracy assessment over time. For this reason, we chose to present the GPL method using a case of mandibular reconstruction with a CAD-CAM titanium patient-specific implant.

While some methods discourage superimposing pre- and postoperative STL models due to reconstruction hardware scattering^8,27,28^, GPL minimizes this issue using an ICP algorithm with *Auto-deviation Elimination*, which excludes erroneous points.

Current methods, including GPL, are limited by image data quality. Preoperative and postoperative CT scans may vary due to different scanners and parameters^8^.

The GPL method, however, focuses on accuracy assessment, not image acquisition improvements, which remain an active research area^29–31^.

Van Baar et al. 2019 suggested starting the alignment from the condylar processes of the mandible on the postoperative STL model^18^. However, the condylar unit is often included in the resection plan, limiting its use for superimposition across the full spectrum of mandibular bone defect reconstructions^20,32,33^. Moreover, the condyle can undergo displacement due to mechanical overload, loss of dental elements, detachment/resection of masticatory muscles, or postoperative factors, such as soft-tissue edema^34–37^. These factors may resolve over time, altering the postoperative anatomy, which complicates long-term accuracy assessment. Current accuracy methods compare early postoperative result (within 1-month post-surgery) to the planned reconstruction^7^, but long-term stability of the reconstruction and the impact of clinical factors on the outcome require further analysis. For this reason, the method should be automated and operator-independent and meet al.l the requirements outlined in the GPL.

Comparing the postoperative and preoperative/planned 3D models usually involves manual selection of anatomical landmarks, which can introduce variability and affect accuracy, especially in the alignment of condylar processes^38^. Manual alignment may lead to inconsistencies, and the final ICP alignment may not always reflect the true anatomical position, complicating clinical decision-making.

Some methods use 3D colorimetric maps to visualize distortions between the planned and postoperative models^17,28,38–40^, but a clear quantitative measure of deviation is missing.

GPL overcomes the limitation of current methods by using roto-translational matrices to quantify 3D spatial deviations, and by employing a unique reference system (GPL-RS) to describe the spatial position and orientation of any model for any patient. This approach relies on the physical-geometric elements of the reference mandible, which are unique and identifiable for each patient, independent of the severity of any mandibular defect. This ensures consistent and reliable measurements, regardless of operator or software variability.

Bevini et al. (2023)^41^ proposed using roto-translational matrices to assess mandibular reconstruction accuracy in 3D. However, their method lacks a standardized reference system, relies on the software’s coordinate system, and involves manual PSI superimposition, all which limit reproducibility and increase measurement uncertainty.

In contrast to these manually-driven approaches, the proposed automated methodology is designed to enhance reproducibility by eliminating operator-dependent variability from the workflow.

The performance of the proposed automated procedure was compared with the manual execution of the same steps. In the assessment of the third roto-translational matrix calculated using both methodologies, the largest rotational deviation was observed in Case 5, where the Rot-X error reached 0.075 degrees. Similarly, the maximum translational error was recorded in Case 5, with the Trans-Z component exhibiting a deviation of 0.105 mm. The discrepancies observed can be attributed to variations in the implementation of the two methodologies. Specifically, the automated procedure utilizes custom-coded functions that may differ from those embedded in the software employed for the manual method. Notable discrepancies primarily arise during the phases of best-fit alignment and global registration. In the automated method, alignment tolerances are calculated automatically by the software and vary according to the clinical case under consideration, typically ranging from 0.90 mm to 1.10 mm. In contrast, the manual procedure utilized a standardized tolerance of 1 mm for all clinical cases examined. This standardization may contribute to the observed differences in both rotational and translational errors, as the adaptability of the automated method to individual cases permits finer adjustments, potentially enhancing overall accuracy.

Future work should extend the validation of the GPL method across to a larger patient cohort, encompassing all types of mandibular defects and a wide range of CAD-CAM reconstruction procedures (e.g., fibula, DCIA, scapula).

Despite the intrinsic objectivity and the comprehensive description provided by the components of the Roto-Translational Matrix (RTM), translating the computed deviations between the planned and postoperative mandible into a meaningful clinical interpretation remains challenging. Accurate clinical interpretation is crucial, as it allows surgeons to identify errors in reconstructive procedures and implement corrective strategies, thereby helping to prevent future mistakes. Enhancing the clinical readability and interpretability of the resulting matrices is therefore an essential task that must be addressed in future research.

In conclusion, the GPL method represents a novel and objective metrological framework for the quantitative assessment of precision in CAD-CAM mandibular reconstructions. Its fully automated implementation offers a standardized and highly reproducible tool that provides directional insights, establishing a robust foundation for improving future surgical outcomes.

## Data Availability

All the data supporting our findings are presented in the paper.
